# Punção Trans Túnel Intra-Atrial Guiada por Imagem Multimodal e Sistema de Mapeamento 3-D com Integração de TC em Doente com Flutter Atrial Pós Cirurgia de Senning

**DOI:** 10.36660/abc.20201267

**Published:** 2021-07-15

**Authors:** Andreia Palma, Pedro A. Sousa, Patrícia V. Silva, António Pires

**Affiliations:** 1 Departamento de Cardiologia PediátricaCentro de Referência de Cardiopatias CongénitasCentro Hospitalar e Universitário de CoimbraCoimbraPortugal Departamento de Cardiologia Pediátrica , Centro de Referência de Cardiopatias Congénitas , Centro Hospitalar e Universitário de Coimbra , Coimbra - Portugal; 2 Departamento de CardiologiaUnidade de Pacing e EletrofisiologiaCentro Hospitalar e Universitário de CoimbraCoimbraPortugal Departamento de Cardiologia , Unidade de Pacing e Eletrofisiologia , Centro Hospitalar e Universitário de Coimbra , Coimbra - Portugal

**Keywords:** Punção transeptal, Cirurgia de Senning, Ablação por cateter, Flutter atrial, Transposição das Grandes Artérias

A complexa anatomia pós-cirurgia de Senning condiciona diversos desafios à ablação por cateter. A abordagem trans túnel intra-atrial permite o acesso ao átrio venoso pulmonar e uma melhor manipulação do cateter. Contudo, nestes doentes, a punção dos túneis intra-atriais pode ser difícil de realizar.

Os autores relatam o caso de uma paciente de sexo feminino, de 29 anos, submetida a cirurgia de Senning por dextro-transposição das grandes artérias. A paciente apresentava episódios recorrentes de flutter atrial refratários à terapêutica antiarrítmica, e recomendou-se a indicação de ablação por cateter.

Tendo em conta que a maioria das arritmias pós-cirurgia de Senning têm origem no átrio venoso pulmonar e que a abordagem retrógrada é de difícil execução, optou-se pela punção do túnel intra-atrial. ^1 - 3^

**Figura 1:**
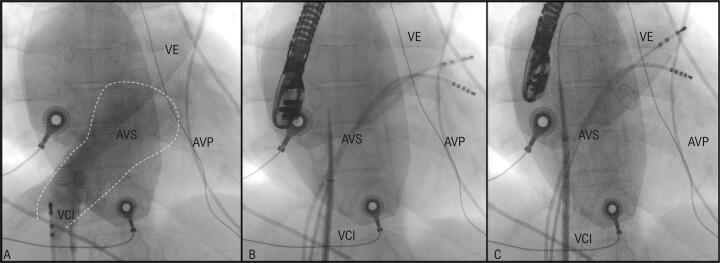
– A: Angiografia na margem inferior do túnel intra-atrial revela a ausência de obstrução ao fluxo, bem como, de fuga intra-atrial. O tracejado delimita os bordos do átrio venoso sistémico (AVS). B e C: Fluoroscopia realizada na incidência póstero-anterior demonstra a punção trans túnel intra-atrial. A agulha transeptal (BRK-1, St. Jude Medical, Inc., Minneapolis, Minnesota) está orientada superiormente e anteriormente (12 horas). A porção superior do túnel intra-atrial está realçada com contraste, permitindo a visualização da ponta da agulha enquanto esta atravessa para o interior do átrio venoso pulmonar (AVP). Posteriormente, uma bainha de 8.5-F (SL0 Swartz braided trans-septal guided, St. Jude Medical, Inc.) é avançada para o PVA. VCI: veia cava inferior; VE: ventrículo esquerdo.

**Figura 2:**
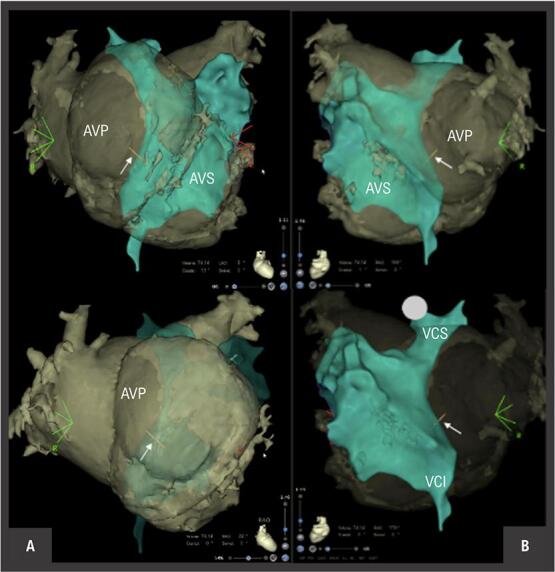
- Mapeamento 3-D (CARTO 3, Biosense Webster, Inc) com integração de imagens de TC dos átrios sistémico (AVS) e pulmonar (AVP). À esquerda (A) encontra-se uma incidência posterior e à direita (B) uma incidência anterior. O túnel intra-atrial está realçado a azul esverdeado. A seta branca demonstra a localização da ponta da agulha aquando da punção trans túnel intra-atrial. VCI: veia cava inferior; VCS: veia cava superior.

**Figura 3:**
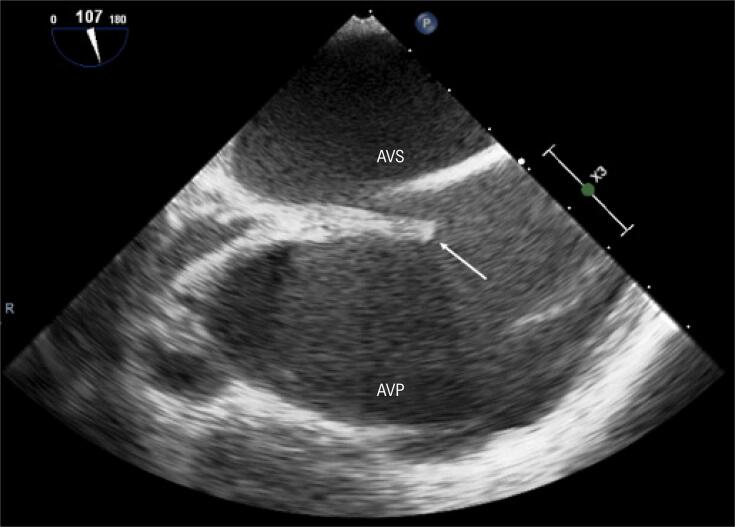
-Ecocardiograma transesofágico que mostra a localização da punção trans túnel intra-atrial. A seta branca aponta para a bainha. AVP: átrio venoso pulmonar; AVS: átrio venoso sistémico.

Realizou-se uma angiografia na margem inferior do túnel intra-atrial ( Figura 1A ). As imagens de tomografia computadorizada (TC) foram integradas com o mapa eletroanatômico do átrio venoso pulmonar (CARTO 3, Biosense Webster) ( Figura 2 ). A agulha transeptal foi conectada ao sistema de mapeamento, o que permitiu a sua visualização em tempo real ( Figura 2 ). A punção do túnel intra-atrial foi realizada sem complicações sob ecocardiograma transesofágico ( Figura 3 ) e fluoroscopia ( Figura 1B e 1C ).

**Figura 4:**
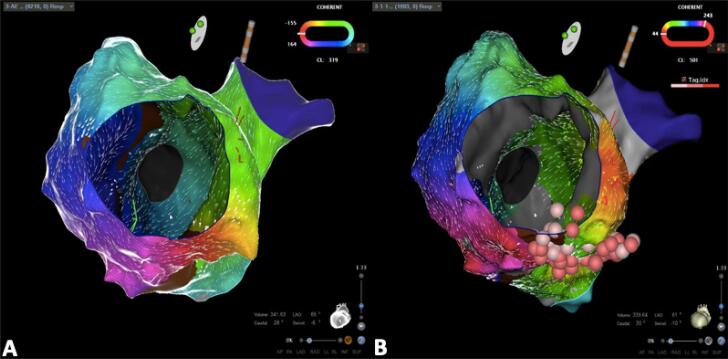
-Mapa de ativação realizado com o algoritmo de mapeamento Coherent e o cateter PentaRay (Biosense Webster, Inc). A – Mapa de ativação do átrio venoso pulmonar com 8216 pontos e uma duração do ciclo de taquicardia de 319 ms; revela um padrão de ativação anti-horário em torno da válvula tricúspide (o vermelho mostra as áreas de ativação mais precoce, enquanto o laranja, amarelo, verde, azul e roxo mostram áreas de ativação progressivamente mais tardias). B – Mapa de ativação do septo posterior do átrio venoso sistémico, realizado com estimulação a 500ms, após ablação por radiofrequência (pontos vermelhos). O mapa inclui 1693 pontos e confirma a presença de bloqueio bidirecional, sem ativação através de ambos os lados atriais do istmo cavo-tricúspide.

O mapa de ativação do átrio venoso pulmonar revelou uma ativação anti-horária em torno da válvula tricúspide (Figura 4A). A aplicação de radiofrequência em ambos os lados atriais do istmo cavo-tricúspide permitiu o término do flutter atrial, com posterior confirmação do bloqueio bidirecional (Figura 4B).

A imagem multimodal e o sistema de mapeamento 3-D com integração de TC permitem a obtenção de referências anatômicas, possibilitando a realização de uma punção eficaz e segura dos túneis intra-atriais, aumentando a probabilidade de sucesso da ablação por cateter em doentes pós-cirúgicos de Senning.
