# Supplementary material for: Punção Trans Túnel Intra-Atrial Guiada por Imagem Multimodal e Sistema de Mapeamento 3-D com Integração de TC em Doente com Flutter Atrial Pós Cirurgia de Senning
Source: Arq Bras Cardiol. 2021 Jul 15;117(1):153–6. [Article in Portuguese] doi: 10.36660/abc.20201267 (PMC8294735; doi:10.36660/abc.20201267)
Supplement: Translation

# Translation: Transbaffle Puncture Using Multimodality Imaging and 3-D Mapping with CT Image Integration in a Patient with Atrial Flutter Post-Senning Procedure

**Authors:** Andreia Palma, Pedro A. Sousa, Patrícia V. Silva, António Pires

**Affiliations:** 1 Portugal Departamento de Cardiologia Pediátrica, Centro de Referência de Cardiopatias Congénitas, Centro Hospitalar e Universitário de Coimbra, Coimbra - Portugal; 2 Portugal Departamento de Cardiologia, Unidade de Pacing e Eletrofisiologia, Centro Hospitalar e Universitário de Coimbra, Coimbra - Portugal

**Keywords:** Transeptal Puncture, Senning Surgery, Catheter Ablation, Atrial Flutter, Arterial Switch Operation

The complex post-surgical anatomy after the Senning procedure presents several challenges for catheter ablation. Transbaffle approach allows access to the pulmonary venous atrium and better catheter manipulation. However, this modified transseptal puncture can be challenging to perform.

The authors present the case of a 29-year-old woman who underwent a Senning procedure for dextro-transposition of the great arteries. The patient had recurrent episodes of atrial flutter despite adequate antiarrhythmic therapy and was referred for catheter ablation.

Since the vast majority of the arrhythmias are related to the pulmonary venous atrium and catheter manipulation through a retrograde approach is difficult, a transbaffle puncture was performed. ^1 - 3^

Angiography in the systemic Senning baffle was first carried out ( Figure 1A ). Images from a CT scan were merged with real-time 3-D electroanatomical mapping of the systemic venous atrium (CARTO 3, Biosense Webster) ( Figure 2 ). The transseptal needle was connected to the mapping system, allowing for the visualization of the needle tip ( Figure 2 ). Under transesophageal echocardiography ( Figure 3 ) and fluoroscopy ( Figures 1B and 1C ), the transbaffle puncture was safely performed.

**Figure 1:** – A: Angiography of the systemic Senning baffle reveals unobstructed flow with no intra-atrial shunting. Dashed line highlights the boundaries of systemic venous atrium (SVA). B and C: Fluoroscopic images, in the postero-anterior view, demonstrating the transbaffle puncture. A transseptal needle (BRK-1, St. Jude Medical, Inc., Minneapolis, Minnesota) is directed superiorly and anteriorly (12 o’clock). Guided by multimodality imaging, the superior portion of the systemic baffle is highlighted and stained with contrast, allowing for visualization of the needle as it crosses into the pulmonary venous atrium (PVA). An 8.5-F sheath (SL0 Swartz braided trans-septal guided, St. Jude Medical, Inc.) is advanced into the PVA. IVC: inferior vena cava; LV: left ventricle.

**Figure 2:** - A 3-D electroanatomical mapping (CARTO 3, Biosense Webster, Inc) of the systemic (SVA) and pulmonary venous atria (PVA), together with CT scan. Left side (A) shows a posterior view and right side (B) an anterior view. The systemic Senning baffle is highlighted in navy green color. The white arrow indicates the needle through the site of the transbaffle puncture. IVC: inferior vena cava; SVC: superior vena cava.

**Figure 3:** -Transesophageal echocardiography, showing the transbaffle puncture site. The white arrow corresponds to the sheath. PVA: pulmonary venous atrium; SVA: systemic venous atrium.

Activation mapping in the pulmonary venous atrium revealed a counterclockwise activation pattern along the tricuspid valve ( Figure 4A ). Radiofrequency was delivered on both sides of the cavotricuspid isthmus, terminating the tachycardia with subsequent confirmation of bidirectional block ( Figure 4B ).

**Figure 4:** - Maps performed with the Coherent mapping algorithm and the PentaRay catheter (Biosense Webster, Inc). A- Pulmonary venous atrium activation map including 8,216 points and 319ms of the tachycardia cycle length, revealing a counterclockwise motion around the tricuspid annulus (red indicates the areas with earliest local activation time, while orange, yellow, green, blue, and purple indicate progressively delayed activation). B- This high-density activation map, performed while pacing at 500ms from the posterior septum of the systemic venous atrium, was performed after radiofrequency delivery (red dots). It included 1,693 points and confirmed the presence of a bidirectional block, with no activation passage through the cavotricuspid isthmus line.

Multimodality imaging and 3-D electroanatomical mapping, together with CT scan provide real-time anatomical landmarks, crucial for an effective and safe transbaffle puncture, increasing the probability of a successful catheter ablation after Senning procedures.
